# The Complexity of the cGAS-STING Pathway in CNS Pathologies

**DOI:** 10.3389/fnins.2021.621501

**Published:** 2021-02-09

**Authors:** Amelia L. Fryer, Amar Abdullah, Juliet M. Taylor, Peter J. Crack

**Affiliations:** Neuropharmacology Laboratory, Department of Pharmacology and Therapeutics, University of Melbourne, Melbourne, VIC, Australia

**Keywords:** STING, neuroinflammation, interferon, central nervous system, cGAS

## Abstract

Neuroinflammation driven by type-I interferons in the CNS is well established to exacerbate the progression of many CNS pathologies both acute and chronic. The role of adaptor protein Stimulator of Interferon Genes (STING) is increasingly appreciated to instigate type-I IFN-mediated neuroinflammation. As an upstream regulator of type-I IFNs, STING modulation presents a novel therapeutic opportunity to mediate inflammation in the CNS. This review will detail the current knowledge of protective and detrimental STING activity in acute and chronic CNS pathologies and the current therapeutic avenues being explored.

## Introduction

Type-I interferons (IFNs) have been strongly implicated in the progression of neuroinflammation in a host of central nervous system (CNS) pathologies including Alzheimer’s disease (Taylor et al., 2014; Minter et al., 2016; Roy et al., 2020), Parkinson’s disease (Main et al., 2016; Qin et al., 2016), traumatic brain injury (Karve et al., 2016; Barrett et al., 2020) and amyotrophic lateral sclerosis (ALS) (Oakes et al., 2017; Shelkovnikova et al., 2019). However, the role of the type-I IFN upstream regulator, the stimulator of interferon genes (STING), in driving this response in the CNS remains largely unknown. Over the last 10 years, STING signalling has been identified as a therapeutic target in autoinflammatory disorders and cancer with its role in neuroinflammation being increasingly recognised. Therefore, a greater understanding of STING signalling in driving a neuroinflammatory response in the diseased brain may also uncover similar therapeutic potential in treating acute and chronic CNS pathologies.

## Type-I Interferon Signalling

The type-I IFN response is known to be a key in the innate immune response to viral infection. However, this response has also been associated with a potent inflammatory response in the absence of pathogen invasion. In the context of viral infection, pathogen-associated molecular patterns (PAMPs) are produced by the invading pathogen and bind to pattern recognition receptors (PRRs) including toll-like receptors (TLR) and cyclic GMP-AMP synthase (cGAS) on the surface of resident immune cells such as microglia and astrocytes in the CNS (Bowman et al., 2003; Olson and Miller, 2004; Jack et al., 2005). This elicits an array of innate anti-viral responses, notably the production of pleiotropic pro-inflammatory cytokines known collectively as the type-I IFNs (Koyama et al., 2008; Murira and Lamarre, 2016). PRRs on CNS immune cells are capable of mounting a similar pro-inflammatory response upon detection of endogenous damage-associated molecular patterns (DAMP) released during injury and stress (Loane et al., 2014; Cox et al., 2015; Kumar, 2019).

Following binding to their cognate receptor, IFNAR (composed of the IFNAR1 and IFNAR2 subunits), the type-I IFNs signal through the Janus kinase (JAK)-signal transducer and activator of transcription (STAT) pathway to elicit an anti-viral, anti-proliferative and immunostimulatory response through interferon-stimulated gene (ISG) induction (Platanias, 2005; Schneider et al., 2014). This results in the secretion of proinflammatory cytokines and chemokines, including tumour necrosis factor-α (TNF-α), interleukin-6 (IL-6), interleukin-1β (IL-1β) and the type-I IFNs themselves (IFN-alpha [IFN-α] and IFN-beta [IFN-β]) (Lousberg et al., 2010).

## The cGas-Sting Pathway

A DNA sensor known as cGAS was recently shown to be critical in type-I IFN induction (Sun et al., 2013). cGAS detects circulating double-stranded DNA (dsDNA) in the cytosol and mounts a potent type-I IFN response through the adaptor protein STING (Sun et al., 2013; Zhang et al., 2013). Exogenous DNA introduced into the cells by invading pathogens is recognised as a PAMP by cGAS, activating STING, a transmembrane adaptor protein located on the endoplasmic reticulum and eliciting a potent type-I IFN response (Li et al., 2013; Watson et al., 2015). Endogenous DNA found outside of the nucleus, in the absence of pathogen invasion, is strongly immunogenic and prompts a pro-inflammatory response, termed sterile inflammation. Released from the nucleus and mitochondria, this DNA can be the result of cell death or genotoxic, mitochondrial or endoplasmic reticulum (ER) stress (Jahr et al., 2001; Kono and Rock, 2008; Petrasek et al., 2013; West et al., 2015; Motwani and Fitzgerald, 2017). This cytosolic DNA is recognised by cGAS as a DAMP and initiates the type-I IFN response through STING (Ishikawa and Barber, 2008; Ishikawa et al., 2009; Sun et al., 2013; Chen et al., 2016b). Once bound to dsDNA, cGAS facilitates the production of a cyclic dinucleotide, 2′5-cyclic adenosine monophosphate guanosine monophosphate (2′5′-cGAMP) from adenosine triphosphate (ATP) and guanosine triphosphate (GTP) (Ablasser et al., 2013); 2′5′-cGAMP is the endogenous agonist of STING, inducing STING phosphorylation and oligomerisation (Ablasser et al., 2013; Shang et al., 2019). Alternatively, STING can be activated by directly binding to bacterial cyclic dinucleotides (CDNs) (Burdette et al., 2011).

**FIGURE 1:**
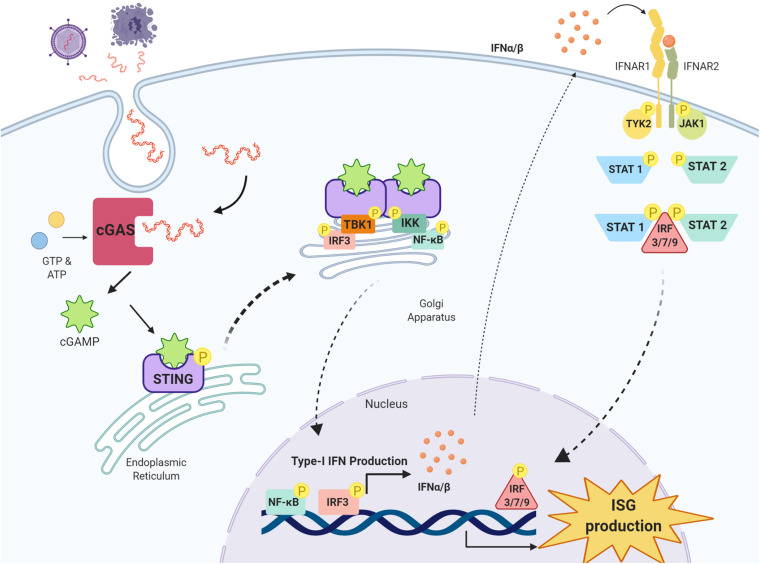
cGAS-STING pathway and type-I IFN signaling. Double-stranded DNA (dsDNA) released from damaged cells or following pathogen infection is taken up cells into the cytosol where it is detected by the enzyme cyclic GMP-AMP synthase (cGAS) which synthesises the cyclic di-nucleotide 2′3′-cGAMP from GTP and ATP. 2′3′-cGAMP is detected by stimulator of interferon genes (STING) residing on the endoplasmic reticulum, and once activated, STING oligomerises and translocates to the Golgi apparatus where it recruits kinases tank binding kinase 1 (TBK1) and IκB kinase (IKK). TBK1 recruits and phosphorylates interferon regulatory factor 3 (IRF3) and IKK recruits and phosphorylates nuclear factor kappa-light-chain-enhancer of activated B cells (NF-κB). IRF3 and NF-κB translocate to the nucleus and upregulate the production of type-I IFNs, which through their receptors interferon alpha and beta receptor subunits 1 and 2 (IFNAR1 and IFNAR2) activate Janus kinase 1 (JAK1) and tyrosine kinase 2 (TYK2). JAK1 and TYK2 activate signal transducer and activator of transcription 1 and 2 (STAT1 and STAT2), which phosphorylate IRF3, IRF7 and IRF9 to stimulate the transcription of interferon stimulated genes (ISG) in the nucleus. Image created with BioRender.com.

Once activated, the STING oligomer translocates to the Golgi apparatus where it recruits and phosphorylates kinases tank binding kinase 1 (TBK1) and IκB kinase (IKK), forming multimeric dimers at the cytosolic domain of STING (Tanaka and Chen, 2012; Liu et al., 2015; Haag et al., 2018; Zhang et al., 2019). Activated STING, TBK1 and IKK recruit and phosphorylate interferon regulatory factor 3 (IRF3) and nuclear factor kappa-light-chain-enhancer of activated B cells (NF-κB) at the C-terminal tail of STING (Tanaka and Chen, 2012; Abe and Barber, 2014; Liu et al., 2015). IRF3 once activated migrates to the nucleus, binds to IFN promoter regions and potently upregulates type-I IFN production (Liu et al., 2015). Following activation by TBK1 and IKK, NF-κB also translocates to the nucleus to upregulate the production of proinflammatory cytokines and chemokines including TNF-α, IL-1β and IL-6, all implicated in driving the neuroinflammatory response in the CNS (Barnes and Karin, 1997) (Figure 1).

## Sting Activity in Viral Infections

Much of our knowledge of STING signalling in the brain originates from mouse models of viral infections. A protective role of STING signalling in mice has been reported following Herpes simplex virus (HSV) and West Nile virus (WNV) infection. Herpes simplex encephalitis (HSE) is a sporadic and fatal form of necrotising encephalitis caused by infection with herpes simplex virus 1 and 2 (Gnann and Whitley, 2017). STING knockout (STING^–/–^) mice demonstrate a markedly increased susceptibility and lethality to HSV-1 infection (Ishikawa et al., 2009). Furthermore, increased HSV-1 viral loads have been detected in the brains of STING^–/–^, STING loss of function (STING^*gt/gt*^) and cGAS knockout (cGAS^–/–^) mice compared to wild-type controls, indicating increased HSE susceptibility (Ishikawa et al., 2009; Reinert et al., 2016). Microglia were the primary producers of the type-I IFNs following HSV-1 infection, and this IFN production was found to be STING dependent (Reinert et al., 2016). STING-deficient mice also display increased morbidity and mortality following WNV infection compared to their wild-type counterparts (You et al., 2013; McGuckin Wuertz et al., 2019). Infection with WNV can progress to West Nile Neuroinvasive Disease (WNND) resulting in meningitis, encephalitis and Parkinsonian-like symptoms (Sejvar et al., 2003). Taken together, these results support a neuroprotective role of STING following HSV-1 and WNV infection.

## Sting Activity in Acute CNS Pathologies

In direct contrast with acute viral infections, STING signalling has recently been shown to be a key instigator of the detrimental prolonged neuroinflammation that ensues following traumatic brain injury (TBI), subarachnoid haemorrhage (SAH) and hypoxia-ischemia (HI) (Table 1). Increased STING signalling was detected in post-mortem human TBI samples (Abdullah et al., 2018) and 24 and 72 h post-CNS injury in mice in a controlled cortical impact model of TBI (Abdullah et al., 2018; Barrett et al., 2020). Additionally, STING^–/–^ mice showed a significantly smaller lesion size compared to wild-type mice suggesting that STING is a driver of TBI-induced neurodegeneration (Abdullah et al., 2018). Sen et al. (2020) identified a possible upstream activator of STING in TBI, a protein produced in response to endoplasmic reticulum stress known as protein kinase R-like ER kinase (PERK). As STING is localised on the endoplasmic reticulum in its resting state, this supports a connection between the ER stress response and STING. Significantly, the TBI-induced activation of STING was attenuated in mice administered a PERK inhibitor (GSK2656157), with reduced lesion volume as well as improvements in anxiety and depression tests reported (Sen et al., 2020). SAH is a form of stroke often resulting from a ruptured aneurism or CNS injury (Tenny and Thorell, 2020). Recently, Peng et al. (2020) found increased STING and p-TBK1 protein expression 12 h post-injury in a mouse model of SAH. The administration of a STING agonist, CMA in SAH mice worsened the neuronal damage and neurobehavioral deficits when compared to vehicle-treated mice. In contrast, administration of a small-molecule STING inhibitor C-176 shortly after SAH modelling conferred neuroprotection by reducing brain oedema, neuronal damage and attenuated the upregulation of pro-inflammatory microglial markers including IL-1β, iNOS and caspase-1 (Peng et al., 2020). Upregulation of STING signalling has also been reported in rats 48–72 h after neonatal HI (Gamdzyk et al., 2020). Furthermore, silencing of STING signalling using siRNA was found to reduce infarct size and neurological impairments 48 h after HI. The significant reduction in TBI lesion size, HI infarct size and neuronal damage through both direct and indirect inhibition of STING signalling suggests a critical role of the STING signalling pathway in perpetuating neurodegeneration with potential therapeutic opportunities to treat acute CNS injuries such as TBI and stroke.

**TABLE 1:** STING activity in acute CNS pathologies.

Pathology	Rodent models	Human	Genetic/pharmacological intervention	References
Traumatic brain injury (TBI)	Increased cortical mRNA and protein expression of STING 24 and 72 h post-TBI in mice	Increased STING mRNA expression reported in post-mortem brain tissue of TBI patients	Smaller TBI-induced lesion size measured in STING^–/–^ mice Small-molecule inhibition of ER-stress protein PERK reduced white matter injury and improved mouse behavioural outcomes by attenuating STING dependent signalling	Abdullah et al., 2018; Barrett et al., 2020; Sen et al., 2020
Subarachnoid haemorrhage (SAH)	Increased STING and p-TBK1 protein expression 12–72 h post-injury in mice	N/A	Administration of small-molecule STING inhibitor C-176 in mice attenuated brain oedema, neuronal injury, and expression of microglial proinflammatory markers and improved neurobehavioral outcomes Administration of STING agonist CMA worsened neurobehavioral performance, exacerbated neuronal damage, and upregulated microglial proinflammatory markers in mice	Peng et al., 2020
Hypoxia-ischemia (HI)	STING upregulated in neonatal rats 24–48 h post-HI	N/A	Inhibition of STING signalling using siRNA attenuated the size of the infarct, neurodegeneration and neurological impairments in rats	Gamdzyk et al., 2020

## Sting Activity in Chronic CNS Pathologies

STING signalling has recently been associated with worsened disease progression in a number of chronic neurodegenerative disease models (Table 2). The ME7 prion disease model is a widely used mouse model for studying chronic neurodegeneration. Nazmi et al. (2019) confirmed STING is a critical driver of the type-I IFN mediated neurodegeneration in this model with mice deficient in STING or IFNAR1 displaying attenuated neuroinflammation (Nazmi et al., 2019). STING has also been reported to exacerbate the neuropathology of a mouse model of Parkinson’s disease (PD). Mutations in *PARKIN*, a ubiquitin ligase, are the most common cause of early-onset PD and have been linked in mouse models to the inefficient removal or autophagy of dysfunctional mitochondria (Pickrell and Youle, 2015). In a model of PD, Parkin^–/–^ mice lacking STING displayed attenuated neuroinflammation and neurodegeneration with improvements in motor function compared Parkin^–/–^ mice (Sliter et al., 2018). This suggests an interplay between mitochondrial stress and STING signalling in PD. Specifically, the inefficient clearing of damaged mitochondria by parkin leads to increased circulating cytosolic mtDNA which when recognised by cGAS initiates the STING signalling cascade. Similarly, a detrimental role for STING in ataxia telangiectasia (AT), an autosomal recessive disorder caused by mutations in the ataxia-telangiectasia (*ATM*) gene, has been reported. AT is clinically characterised by cerebellar degeneration, telangiectasia and immunodeficiency (Amirifar et al., 2019). Mutations in the *ATM* gene in mice have been associated with the accumulation of DNA in the cytoplasm, leading to increased type-I IFN production through a STING-mediated pathway (Hartlova et al., 2015). However, the implications of targeting this STING-mediated IFN production in terms of reducing the cerebellar degeneration and improving motor control in this mouse model are still unknown. Recently, Sharma et al. (2020) identified upregulated cGAS-STING signalling in Huntington’s disease (HD). Increased cGAS protein expression was found in human HD striatal neurons and in neurons derived from Hdh^*Q*111/Q111^ mice. Furthermore, increased expression of p-TBK1 and p-STING, downstream of cGAS was detected in the striatal neurons of the HD mice (Sharma et al., 2020). Increased mRNA levels of *Ccl5* and *Cxcl10* was found to be cGAS dependent in both human and mouse striatal HD tissue further implicating the cGAS-STING pathway in driving the neuroinflammatory response in HD (Sharma et al., 2020). A relationship between STING and TDP-43, a hallmark protein of ALS has recently been established with TDP-43 found to trigger mtDNA release into the cytoplasm, activating the cGAS-STING pathway (Yu et al., 2020). Increased cGAS and cGAMP, the STING activating molecule produced by cGAS was detected in spinal cords and in the cortex of ALS mice overexpressing TDP-43 (Prp-TDP-43^*Tg/+*^). When STING was genetically deleted from these mice, the average lifespan increased by 40% and the mice exhibited improved rotarod performance compared to Prp-TDP-43^*Tg/+*^ mice with intact STING (Yu et al., 2020). Furthermore, elevated levels of the STING activator cGAMP were detected in spinal cord samples from ALS patients (Yu et al., 2020). Together these findings implicate the cGAS-STING pathway in driving the damaging inflammatory processes present in ALS.

**TABLE 2:** STING activity in chronic CNS pathologies.

Pathology	Rodent models	Human	Genetic/pharmacological intervention	References
Parkinson’s disease (PD)	STING pathway implicated in the onset of neuroinflammation, neurodegeneration and motor deficits in Parkin^–/–^ mice	N/A	Genetic deletion of STING attenuates the loss of dopaminergic neurons and motor deficits seen in Parkin^–/–^ mice	Sliter et al., 2018
Ataxia telangiectasia (AT)	STING drives type-I IFN induction in ATM^–/–^ mice	N/A	Genetic deletion of STING reduced type-I IFN response caused by loss of ATM gene	Hartlova et al., 2015
Huntington’s disease (HD)	Increased cGAS, p-TBK1 and p-STING expression found in Hdh^*Q*111/Q111^ mice	Increased cGAS protein expression found in HD striatal neurons	N/A	Sharma et al., 2020
Multiple Sclerosis (MS)	STING-induced type-I IFN production attenuates EAE pathology Mice lacking functional STING display attenuated EAE development	cGAS and STING gene expression is downregulated in relapsed MS patients	Use of STING agonist c-di-GMP delayed disease onset and severity in EAE mouse model Activating STING using antiviral therapeutic ganciclovir was able to attenuate disease progression in EAE mice	Lemos et al., 2014; Mathur et al., 2017; Masanneck et al., 2020
Systemic lupus erythematosus (SLE)	Loss of STING accelerates mortality and disease progression in Lupus prone mice (MRL-Fas^*lpr*^)	ISG inducing activity of sera derived from SLE patients is STING dependent	N/A	Sharma et al., 2015; Kato et al., 2018
Amyotrophic lateral sclerosis (ALS)	Increased cGAS and cGAMP detected in the spinal cords of Prp-TDP-43^*Tg/+*^ mice Genetic deletion of STING in Prp-TDP-43^*Tg/+*^ mice increased average lifespan by 40%	Elevated levels of cGAMP detected in the spinal cords of ALS patients	Administration of small-molecule STING inhibitor H-151 reduced cortical and spinal cord proinflammatory cytokine gene expression and reduced neurodegeneration in ALS mice. Death of ALS patient iPSC -derived motor neurons was prevented following H-151 administration	Yu et al., 2020

In contrast to other chronic neurodegenerative diseases, the type-I IFNs have been implicated in a neuroprotective role in Multiple Sclerosis (MS). Intramuscular IFN-β1 is used therapeutically in patients with relapsing and remitting MS to reduce the number and volume of brain lesions; however, its exact mechanism of action remains to be fully elucidated (Kieseier, 2011). More recently, a protective effect of STING activation has been reported in experimental autoimmune encephalitis (EAE), a mouse model of MS. The CDN STING activator c-di-GMP and DNA nanoparticles (DNPs) were able to delay EAE and reduce the overall disease severity (Lemos et al., 2014). A later study confirmed the therapeutic potential of activating STING in EAE mice using a clinically approved antiviral, ganciclovir (GCV), which has been previously shown to attenuate EAE pathology in mice. The study found that STING was required for GCV to elicit its neuroprotective and anti-inflammatory activity in EAE mice (Mathur et al., 2017). This is supported by a gene expression analysis of peripheral blood mononuclear cells from relapsing remitting MS patients and healthy donors. cGAS and STING gene expression was downregulated in relapsed MS patients compared to both patients in remission and healthy donors, further suggesting a neuroprotective role of cGAS-STING signalling in MS (Masanneck et al., 2020). Together these results support the therapeutic potential of upregulating STING mediated-type-I IFN production through the use of STING agonists as an adjunct to antivirals such as GCV used in MS. Although upregulating IFN production through STING shows promise in mouse models of MS, this same efficacy may not translate to humans. Long-term IFN-β therapy has been associated with the increased incidence of adverse CNS effects including depression and ‘flu-like symptoms’ such as fever, muscle aches and headaches, which have been noted as a major factor for MS patients to discontinue IFN-β therapy in addition to poorly perceived efficacy (Neilley et al., 1996; O’Rourke and Hutchinson, 2005; Fox et al., 2013).

The role of STING in systemic lupus erythematosus (SLE) is also less clear, with both detrimental and beneficial effects in the disease progression reported. SLE is an autoimmune disease that can present as neuropsychiatric lupus (NPSLE) in approximately 20% of cases causing a range of syndromes including aseptic meningitis, cerebrovascular disease, seizure disorders and cognitive dysfunction (Manson and Rahman, 2006). Lupus prone mice (MRL-Fas^*lpr*^) lacking STING display an accelerated disease progression and mortality compared to lupus-prone mice (Sharma et al., 2015). However, a later study with apoptosis-derived vesicles (AdMVs) from the sera of patients with SLE identified dampened ISG induction in STING^–/–^ reporter cells compared to parental cells when challenged with these AdMVs (Kato et al., 2018). This suggests that ISG induction in human SLE is amplified in the presence of STING. The contrasting roles of STING in promoting disease susceptibility and severity whilst amplifying the autoinflammatory response in SLE warrant further investigation. In addition, these studies failed to study the role STING may play in the CNS and lupus progression.

## Sting Activity in the Ageing Brain

Ageing is a major risk factor for the development of neurodegenerative diseases. Cell senescence is a hallmark feature of ageing as senescent cells perpetuate chronic low-level inflammation by adopting the senescence-associated secretory phenotype (SASP), resulting in the release of proinflammatory cytokines, chemokines, growth factors and extracellular matrix proteins (Coppé et al., 2010; McHugh and Gil, 2018). Increasing evidence has implicated cGAS-STING signalling in the chronic inflammation associated with age-induced cell senescence. Increased cytosolic DNA has been found in aged diploid fibroblasts when compared to younger cells (Lan et al., 2019). The inflammation observed in these cells was cGAS-STING dependent, suggesting aging triggers cGAS-STING mediated inflammation through the accumulation of cytosolic DNA (Lan et al., 2019). This idea was further supported using murine embryonic fibroblast cells, where genetic deletion of cGAS attenuated senesce processes in these cells (Glück et al., 2017). Furthermore, upregulation of proinflammatory markers IL-6 and CXCL-10 following irradiation *in vitro* and *in vivo* was found to be dependent on intact cGAS-STING signalling (Glück et al., 2017). Further supporting cGAS-STING signalling as a driver of age-induced inflammation, cells from AT and Hutchinson-Gilford progeria patients have been shown to have increased cytosolic DNA compared to healthy donor cells (Lan et al., 2019). AT and Hutchinson-Gilford progeria are genetically inherited disorders characterised by premature ageing (Merideth et al., 2008; Rothblum-Oviatt et al., 2016). Together these findings implicate cGAS-STING signalling as a driver of detrimental chronic inflammation associated with ageing.

## Therapeutic Potential of Targeting Sting

IFN-α and IFN-β are abundantly expressed and highly implicated in normal and pathological conditions (González-Navajas et al., 2012; Malireddi and Kanneganti, 2013; Cho and Kelsall, 2014). As a result, targeting type-I IFN signalling has shown to be promising in the treatment of infectious diseases, various cancers, and autoimmune diseases including multiple sclerosis, SLE and psoriasis (Di Domizio and Cao, 2013; Aricò et al., 2019). However, their therapeutic success in clinical trials have been variable and is severely limited due to side effects which include fever, cognitive dysfunction, depression and in some cases death. With mounting evidence implicating the cGAS-STING pathway in driving neuroinflammation in both acute and chronic neurological diseases, modulation of type-I IFN signalling by targeting cGAS-STING pathways represents a viable therapeutic in the treatment of CNS disorders.

**FIGURE 2:**
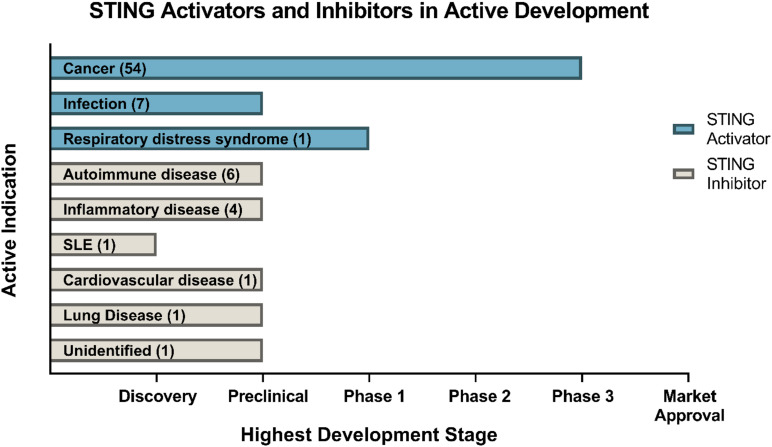
Drugs targeting STING currently in development. Data obtained through Cortellis search (Clarivate Analytics). Data correct as of December 2020.

The development of small-molecule agonists and antagonists to target STING signalling in mice and humans is a growing area of research (Table 3). Small-molecule agonists and CDN analogues are currently being developed, with several in phase I and II clinical trials for use against viral infections such as human papillomavirus (HPV) and in cancer immunotherapy with a focus on solid tumours (Figure 2) (Feng et al., 2020; Zhang et al., 2020). The use of bacterial and synthetic CDNs such as c-di-GMP and 3′3′cGMP as vaccine adjuvants have displayed promising anti-tumour activity in mouse models of metastatic breast cancer, melanoma and colon carcinoma (Chandra et al., 2014; Fu et al., 2015). STING activating nanoparticles is an increasingly active area of research in treating solid tumours as nanoparticles may be able to overcome the translational challenges of using CDNs, which include their negative charge and susceptibility to being rapidly enzymatically degraded (Wilson et al., 2018; Luo et al., 2019; Su et al., 2019).

**TABLE 3:** STING modulators.

Compound	Affinity	Disease model	Disease outcome	References
STING activator CDNs	mSTING; Kd∼110 nM, hSTING; ∼4.59 nM	Viral infection^+^; Cancer^+^; EAE^+^	Protective against viral infections; shows anti-tumour activity in mouse models of various cancers; delays EAE and reduce the overall disease severity	Burdette et al., 2011; Chandra et al., 2014; Lemos et al., 2014; Li et al., 2014
STING activator DMXAA	mSTING; Kd∼130 nM	Cancer^+^	Shows potent anti-tumour activity in mouse models of lung cancer and mesothelioma	Conlon et al., 2013; Kim et al., 2013
STING activator CMA	mSTING; Kd:3.5 μM	SAH^–^	Exacerbates neuronal damage and neurobehavioral deficits in a mouse model of SAH	Zhang et al., 2015; Peng et al., 2020
STING activator GCV	m/hSTING; Kd:N/A	EAE^+^; Viral infection^+^	Attenuates EAE pathology in mice; protective against cytomegalovirus infections	Mathur et al., 2017
STING inhibitor GSK2656157	N/A	TBI^+^	Reduces lesion volume and improves neurobehavioral outcome	Sen et al., 2020
STING inhibitor C-176	mSTING; IC_50_ < 50 nM	AGS^+^; SAH^+^	Ameliorates STING associated-inflammation in AGS mouse model; reduces brain oedema, neuronal damage and neuroinflammatory response in SAH mouse model	Haag et al., 2018; Peng et al., 2020

*Kd (dissociation constant) indicates the affinity of STING binding to the compound ligand. Half-maximal inhibitory concentration (IC_50_) is the concentration of an inhibitor where the response (or binding) is reduced by half. ^(+)^ denotes beneficial and ^(–)^ denotes detrimental effects of STING compound in disease outcome. mSTING, mouse STNG; hSTING, human STING.*

Currently, there is limited research on the use of STING agonists and antagonists in the CNS. Nonetheless, STING activation in astrocytes has been reported to promote the growth of brain metastatic cancer cells in mice (Chen et al., 2016a). This study found that brain metastatic cells co-cultured with astrocytes transported cGAMP into the neighbouring astrocytes via *Cx43* gap junctions to activate STING. Astrocytic production of IFN-α and TNF-α was correlated with the inhibition of apoptosis in the metastatic brain cells when exposed to chemotherapy, suggesting that STING activation in astrocytes promotes survival of brain metastatic cancer cells in mice (Chen et al., 2016a). Further studies elucidating the impact of STING activation and the development of brain cancers will be required to assess the suitability of STING agonists for the treatment of these CNS pathologies.

STING inhibitors in the form of competitive antagonists and covalent inhibitors have also been developed to treat autoinflammatory conditions such as Aicardi Goutières syndrome (AGS) and STING-associated vasculopathy with onset in infancy (SAVI) (Haag et al., 2018; Hansen et al., 2018). These compounds are nitrofuran and nitro-fatty acid derivatives and have shown promising results in reducing serum type-I IFN concentrations in a Trex1^–/–^ mouse model of AGS and SAVI patient-derived fibroblasts (Haag et al., 2018; Hansen et al., 2018). C-176 was shown by Haag et al. (2018) to ablate STING activity through the blockade of activation-induced palmitoylation, impeding STING’s ability to translocate to the Golgi in response to CDN binding. Administration of C-176 intraperitoneally in SAH induced mice has also been shown to improve neurobehavioural outcomes and reduced the expression pro-inflammatory microglial markers including IL-1β, TNF-α and IL-6 (Peng et al., 2020). Peng et al. (2020) reported that inhibition of adenosine monophosphate-activated protein kinase (AMPK), a regulator of cellular energy homoeostasis (Hardie, 2011) reversed the anti-inflammatory activity of C-176 *in vitro* and *in vivo*, suggesting that AMPK has a role in the inhibition of STING through C-176.

Gain of function mutations in the STING gene, *TMEM173*, is associated with autoinflammatory disorders including familial chilblain lupus and STING-associated vasculopathy with onset in infancy (SAVI). A heterozygous mutation in *TMEM173* has been linked to familial chilblain lupus, a rare autoinflammatory pathology characterised by early onset arthralgia and lymphopenia (König et al., 2017). This gain of function mutation enhanced the ability of STING to dimerise in the absence of cGAMP, resulting in constitutive IFN activation (König et al., 2017); *de novo* germline mutations in STING have been identified in SAVI patients, causing a hypersensitivity to ligand activation in STING, resulting in constitutive production of type-I IFNs (Liu et al., 2014). This mutation manifested clinically in the onset of systemic inflammation, acral necrosis and interstitial lung in infants and children (Liu et al., 2014).

Multiple STING allele variants have been detected in the human population, with one variant *R293Q*, known to impair the function of STING (Patel and Jin, 2019). A multicentre study carried out in Polish Caucasians over 65 years of age, found individuals carrying the *R293Q* STING allele were less susceptible to age-related chronic lung disease due to the lowered immune sensitivity associated with the dysfunctional STING (Hamann et al., 2019). Given that mutations in the STING gene correlate with disease prognosis, genetic screening for STING mutants may serve as potential biomarker in diseases linked to impaired STING function. In addition, changes in expression levels of molecules downstream in the STING pathway have also been associated with various diseases. Exome sequencing has identified TBK1 as a risk factor in ALS and fronto-temporal dementia with mutations in the human TBK1 gene implicated in neuroinflammatory disorders (Cirulli et al., 2015; Ahmad et al., 2016; Wilke et al., 2017). Unregulated levels of IRF3 and the type-IFNs have been implicated in tumorigenesis and progression of autoimmune disorders including rheumatoid arthritis (RA), SLE and primary Sjogren’s syndrome (Gottenberg et al., 2006; Crow, 2014; Muvaffak et al., 2014; Jiao et al., 2018; Barrat et al., 2019; Petro, 2020). Together these results implicate genetic alterations in STING and its downstream mediators in the progression of autoinflammatory disorders and highlight the importance of genetic characterisation of STING to gain a deeper insight into the mechanisms of STING dysregulation in neurodegeneration.

Key challenges in targeting STING include the high heterogeneity of STING in the human population and the differences in structure and signalling between mouse and human STING. There are multiple alleles present for the gene that encodes STING (*TMEM173*) and these alleles have been found to exhibit a high degree of population stratification (Patel et al., 2017). Markedly different *TMEM173* genotypes have been detected in different ethnic groups and differential STING protein expression has been found in cells of these different genotypes (Patel et al., 2017). Further characterisation of the variants of STING present in different populations is required to ensure the accurate development of STING compounds. The structural difference in mice (mSTING) and humans (hSTING) is also a challenge in STING targeted therapies and has been attributed to the clinical failure of the small-molecule STING activator DMXAA. This initially displayed promising anti-tumour capability in mice but failed to translate to human studies due to the compound’s inability to bind to hSTING (Conlon et al., 2013). This highlights the importance of developing animal and cell culture models that can accurately mimic the activity of hSTING with the binding capabilities of the compound with hSTING tested. The use of rats instead of mice has also been proposed to be a more accurate model to study compounds targeting STING as rat STING (rSTING) has been found to mimic the substrate binding properties of hSTING more so than mSTING (Zhang et al., 2015). Moreover, the double-edge sword of the immune system in suppressing and promoting tumour growth poses a challenge in targeting STING as a cancer therapy. Prolonged activation of STING can result in a tolerogenic immune response, chronic neuroinflammation, increased tumour growth and impaired T-lymphocyte function all of which are detrimental in cancer treatment (Huang et al., 2013; Ahn et al., 2014; Larkin et al., 2017; Lemos et al., 2020). Conversely, prolonged suppression of the neuroinflammatory response by STING inhibition may be detrimental in the treatment of diseases that require an acute, beneficial initial neuroinflammatory response as seen in spinal cord injury, stroke, and traumatic brain injury (DiSabato et al., 2016; Simon et al., 2017; Shields et al., 2020). Given the multifaceted role of STING and the magnitude of STING signalling pathways still remains to be determined, caution should be taken in the development and application of STING modulators. The optimal therapeutic window for STING activation and inhibition will be essential in allowing STING modulators to exert their protective effects whilst minimising toxicity in any disease treatment.

## Conclusion

Recent studies on STING signalling in the brain have increased our understanding of the role of this pathway in neural innate immunity and inflammation-mediated neurodegeneration. STING activation occurs in response to a wide array of stressors, from viral infection to ER and mitochondrial stress, suggesting it is a major player in a number of neuropathologies. With both beneficial and detrimental effects of STING reported, it appears there will be complexity in targeting this pathway. However, with multiple small-molecule agonists and antagonists of STING emerging and the critical validation of findings from mouse models in humans, we are gaining an increased understanding of the therapeutic potential of targeting STING in specific CNS disorders.

## Author Contributions

ALF, AA, JMT, and PJC contributed to the writing of this manuscript. All authors contributed to the article and approved the submitted version.

## Conflict of Interest

The authors declare that the research was conducted in the absence of any commercial or financial relationships that could be construed as a potential conflict of interest.
